# Development and validation of a novel UPLC-Q-Orbitrap HRMS method for determining p-Nitroaniline and its metabolites in blood

**DOI:** 10.3389/fchem.2025.1631203

**Published:** 2025-10-08

**Authors:** Peng Wang, Junlin Wang, Qiuliang Xu, Hua Zou, Xiangjing Gao, Hong Ren, Yiyao Cao

**Affiliations:** Zhejiang Provincial Center for Disease Control and Prevention, Hangzhou, China

**Keywords:** occupational exposure, p-Nitroaniline, blood, UPLC-Q-Orbitrap HRMS, metabolite

## Abstract

An accurate, reliable, and sensitive method was successfully developed to simultaneously determine p-Nitroaniline (p-NA) and its metabolites in blood based on the hyphenated technology of quadrupole-orbitrap high resolution mass spectrometry (Q-Orbitrap HRMS) and ultra-performance liquid chromatography (UPLC). After the blood sample was extracted with ethyl acetate, the organic layer was taken for injection and analysis. Within the concentration range of 1–100 μg/L, the calibration curves of p-NA and its metabolites had a good linearity, with correlation coefficient(*r*) values greater than 0.999. This method has excellent precision and accuracy. The intra- and inter-day coefficients of variation (CVs) were less than 9.9% and 8.7% respectively, and the analytical accuracy ranged from 83.1% to 101.3%. The lower limits of detection (LLODs) for all target analytes were between 0.6 μg/L and 2.2 μg/L, and the lower limits of quantification (LLOQs) were between 2.0 μg/L and 7.4 μg/L. In addition, the application potential of this method was verified by analyzing the blood samples of workers exposed to p-NA. The results indicated that this method was accurate, reliable and sensitive, and was applicable to the detection of p-NA and its metabolites in the blood.

## 1 Introduction

p-Nitroaniline(p-NA), as an important chemical raw material, is widely used in many industries such as dyes, pesticides, imaging, and pharmaceuticals (Chai et al., 2018; Ma et al., 2009; Sun et al., 2007). China holds a significant position in the global p-NA field and is both a major producer and a major consumer. As of 2020, the global market demand for p-NA reached 129,200 tons. China’s production capacity was approximately 110,000 tons per year, and the annual consumption in the domestic market was about 90,000 tons (Huajing Industrial Research Institute, 2021). Such a large production capacity and consumption volume mean that there are a relatively large number of workers in China who are occupationally exposed to p-NA, which makes the occupational exposure risk at a relatively high level.

p-NA can easily mix with sweat and be absorbed through the skin, and its dust and vapor can be inhaled through the respiratory tract (National Health Commission of the People’s Republic of China, 2019; Bronaugh and Maibach, 1985). p-NA is a potent methemoglobin former (French et al., 1995). In cases of acute poisoning, patients may exhibit symptoms such as cyanosis, fatigue, nausea, dizziness, chest tightness, vomiting, shortness of breath, disturbance of consciousness, arrhythmia (Gu and Bao, 2011; Zhang et al., 2006). Additionally, it can cause the destruction of red blood cells, leading to hemolytic anemia. The methemoglobin and its decomposition products released after the destruction of red blood cells can also damage the liver and kidneys (Ministry of Environmental Protection of the People’s Republic of China, 2009; Xia, 1991). The American Conference of Governmental Industrial Hygienists has listed it as a potential occupational carcinogen that can be absorbed through the skin (American Conference of Governmental Industrial Hygienists, 2021). Therefore, occupational exposure to p-NA may be associated with serious health risks.

**FIGURE 1:**
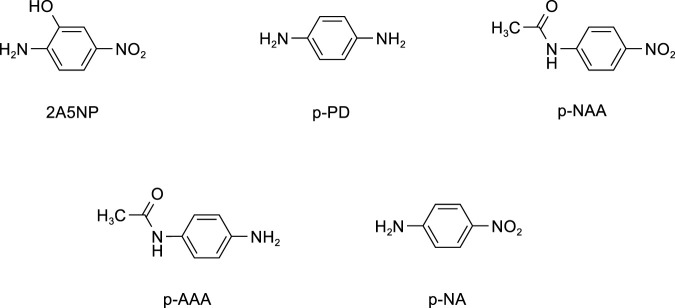
The chemical structures of p-NA and its metabolites.

Internal exposure assessment is a means to effectively control the occupational health risks of p-NA. It can comprehensively and accurately reflect the actual degree of the body’s exposure to p-NA. Specifically, by detecting p-NA and its metabolites in human blood, the internal exposure level of an individual can be precisely evaluated. Therefore, developing an accurate, reliable, and sensitive identification and analysis method for p-NA and its metabolites in blood is an indispensable prerequisite for internal exposure assessment. Relevant research has shown that when a large amount of p-NA enters the body, multiple substances can be detected in the blood. These compounds encompass 2-amino-5-nitrophenol (2A5NP), which is produced via hydroxylation of the benzene ring; p-phenylenediamine (p-PD), resulting from nitro reduction; p-nitroacetanilide (p-NAA) and p-aminoacetanilide (p-AAA), both formed through acetylation of amino group; and unaltered p-NA (Bakdash et al., 2006). The structural formulas of all the compounds are shown in Figure 1.

As of now, there have been no reports of a systematic quantitative analysis method specifically for p-NA and its metabolites in blood. In the limited research materials, the relevant determination method was only briefly mentioned in a report on a p-NA poisoning incident. In this report, the researchers used derivatization combined with gas chromatography-mass spectrometry and high performance liquid chromatography to determine p-NA and its metabolites (Bakdash et al., 2006). Unfortunately, this report lacks detailed information on a series of key technical indicators such as accuracy, precision, sensitivity, and matrix effect, which limits the scientific nature of the method. In addition, the source of the samples tested by this method also has limitations. All samples were taken from patients with acute p-NA poisoning, in whose blood the concentrations of p-NA and its metabolites are usually at a relatively high level. In an actual working environment, most workers are only exposed to low concentrations of p-NA. Due to the huge difference in exposure doses, the sample situation of acute poisoning patients may be significantly different from the actual situation of low-exposure workers. Therefore, it is currently unclear whether the method in this report can be accurately and effectively applied to the biological monitoring of these low-exposure workers in a normal working environment, and further research and verification are needed.

In this research work, we were committed to developing an analytical method that could accurately and simultaneously determine p-NA and its metabolites in the blood of workers with low-level exposure by means of ultra-performance liquid chromatography-quadrupole-orbitrap high resolution mass spectrometry (UPLC-Q-Orbitrap HRMS), filling the gap in this field. The Orbitrap mass analyzer has very high resolution, and its mass accuracy is far superior to that of triple quadrupole mass spectrometry. Therefore, its qualitative accuracy is also much higher than that of triple quadrupole mass spectrometry, which can eliminate the interference of matrix components with similar mass numbers on the target analytes (Abou-Elwafa Ab et al., 2019; Enders et al., 2022; Qi et al., 2019; Zhao et al., 2019). In addition, the Orbitrap mass analyzer has an extremely fast scanning speed. This characteristic ensures that, under high-resolution conditions, the instrument will not be affected by a lack of scan points, thereby guaranteeing sufficient sensitivity and achieving quantitative performance comparable to that of triple quadrupole mass spectrometry (Li et al., 2017).

## 2 Materials and methods

### 2.1 Standards and reagents

The standards, including p-NA (99.9%), p-NA-D_4_ (99.9%), p-PD (99.9%), p-PD-D_4_ (99.3%), p-NAA (99.9%), p-NAA-D_4_ (99.9%), 2A5NP (99.7%), 2A5NP-D_3_ (≥95.9%), p-AAA (99.9%) and p-AAA-D_4_ (99.3%), came from Alta Scientific Co., Ltd. HPLC-grade methanol came from Fisher Scientific (United States), HPLC - grade ethyl acetate came from Tedia (United States), and HPLC-grade formic acid came from Shanghai Macklin Biochemical Co., Ltd.

### 2.2 UPLC-Q-Orbitrap HRMS instrumentation and setting

The equipment was purchased from Thermo Scientific and consisted of one Vanquish UPLC and one Orbitrap Exploris 120 equipped with a heated electrospray ionization source. Chromatographic separation was carried out on an ACQUITY UPLC®BEH Shield RP18 column (2.1 mm i. d. × 10cm, 1.7 µm particle size, Waters). The column temperature was kept at 30 °C. The injection volume was 5 µL. In mass spectrometry, sheath gas was 35 arb, aux gas was 7 arb, ion transfer tube temperature was 320 °C and vaporizer temperature was 275 °C.

The mobile phase consisted of aqueous phase (solvent A: 0.1% formic acid aqueous solution) and organic phase (solvent B: methanol), following a gradient elution program: 0–1.5 min, 5% B; 1.5–2.5 min, 5%–60% B; 2.5–7.0 min, 60%–80% B; 7.0–7.5 min, 80%–5% B; 7.5–10 min, 5% B. The total run time was 10 min with a constant flow rate of 200 μL/min. Mass spectrometry analysis was carried out using the “tMS^2^” mode, which is similar to the parallel reaction monitoring mode. Isolation window (m/z) was 1.0, orbitrap resolution was 15000@m/z 200. Table 1 presents the mass spectral parameters.

**TABLE 1:** Mass spectometry parameters.

Compound	T-start to T-stop (min)	Precursor ion (m/z)	Secondary mass spectrum scan range (m/z)	Extracted ion (quantitative ion) (m/z)	Collision energy (V)	Ionization form	Spray voltage (V)
p-PD	0–1.2	109.0758	40.0000–131.7673	92.0494	15	Positive	3,500
p-PD-D_4_	0–1.2	113.1010	40.0000–135.8730	96.0745	15	Positive	3,500
p-AAA	1.2–3.0	151.0864	40.0000–174.6810	109.0759	15	Positive	3,500
p-AAA-D_4_	1.2–3.0	155.1117	40.0000–178.7239	113.1010	15	Positive	3,500
2A5NP	3.0–6.6	153.0301	40.0000–176.6007	122.0249	15	Negative	2,500
2A5NP-D_3_	3.0–6.6	156.0493	40.0000–179.6803	125.0436	15	Negative	2,500
p-NA	3.0–6.6	139.0502	40.0000–162.3412	122.0474	15	Positive	3,500
p-NA-D_4_	3.0–6.6	143.0756	40.0000–166.4471	126.0725	15	Positive	3,500
p-NAA	6.6–7.5	179.0455	40.6273–203.1364	137.0357	15	Negative	2,500
p-NAA-D_4_	6.6–7.5	183.0702	41.4483–207.2416	141.0608	15	Negative	2,500

### 2.3 Stock solution preparation

5.0 mg of each standard, including p-PD, p-AAA, 2A5NP, p-NA, and p-NAA, were accurately weighed. These weighed standards were then dissolved in methanol, and the resulting solution was transferred into a 10-mL volumetric flask. Subsequently, the volume was adjusted to the calibration mark with methanol to formulate a mixed standard stock solution having a concentration of 500 μg/mL.

Likewise, 5.0 mg of each isotope internal standard, including p-PD-D_4_, p-AAA-D_4_, 2A5NP-D_3_, p-NA-D_4_, and p-NAA-D_4_, were accurately weighed. These weighed isotope internal standards were then dissolved in methanol, and the resulting solution was transferred into a 10-mL volumetric flask. Subsequently, the volume was adjusted to the calibration mark with methanol to formulate a mixed isotope internal standard stock solution having a concentration of 500 μg/mL.

The mixed isotope internal standard stock solution and the mixed standard stock solution should be stored in a dark environment at a temperature of −20 °C.

### 2.4 Calibration solution preparation

The mixed standard stock solution and the mixed isotope internal standard stock solution underwent dilution with ethyl acetate. This dilution process was carried out to formulate a series of mixed standard solutions. In these solutions, the concentrations of each individual component were precisely set at 1 μg/L, 5 μg/L, 10 μg/L, 50 μg/L, 100 μg/L, and 150 μg/L. Each of the prepared mixed standard solutions was spiked with the corresponding isotope internal standard at a concentration of 20 μg/L.

### 2.5 Sample treatment procedures

0.4 mL of the blood sample was transferred into a 10.0 mL centrifuge tube equipped with a stopper, then 12 μL of 5 μg/mL mixed isotope internal standard solution, which was obtained by diluting the mixed isotope internal standard stock solution with a concentration of 500 μg/mL with water, was added to the blood sample. Subsequently, 3.0 mL of ethyl acetate was introduced, and the mixture was subjected to vigorous shaking for 5 min to facilitate extraction. After standing still for 5 min, approximately 1.0 mL of the supernatant was then filtered through a 0.22 μm glass-fiber syringe filter in preparation for analysis. When the concentration of the sample exceeded the upper limit of the linear range, dilution was required before measurement.

### 2.6 Method validation

The method validation was meticulously performed in strict accordance with the “Bioanalytical Method Validation-Guidance for Industry” issued by U.S. Department of Health and Human Services Food and Drug Administration (United States Food and Drug Administration, 2018). Comprehensive validation was carried out for multiple crucial parameters, including linearity, sensitivity, selectivity, carryover, precision, accuracy, matrix effect, extraction recovery, and the stability of the analyte.

#### 2.6.1 Linearity and sensitivity

A matrix-free calibration curve consisting of six concentration levels (with internal standard added) was adopted to evaluate the linearity of each target analyte. The sensitivity was evaluated using the LLOD and LLOQ. According to the guidelines for the validation of analytical method in Chinese Standards (National Health and Family Planning Commission of the People’s Republic of China, 2017), the LLOD and LLOQ were established by performing ten replicate analyses of low-concentration samples spiked at the LLOQ level. The specific calculation formulas are as follows:

$$\text{LLOD}=K\times 3\times S\times \frac{c}{b}$$ (1)

$$\text{LLOQ}=K\times 10\times S\times \frac{c}{b}$$ (2)

Where: *K* is the volume conversion factor for sample pretreatment; *S* is the standard deviation of the response values from 10 repeated measurements; *c* is the known concentration of the analyte at a low level; and *b* is the response signal corresponding to *c*.

#### 2.6.2 Selectivity and carryover

Blank blood samples from six different individuals were mixed and then analyzed to determine the selectivity of the target analytes and isotope internal standard. To determine whether the analytes in the sample would remain in the instrument and affect subsequent sample analysis, we conducted a carryover test. A high-concentration calibration solution was injected into the instrument, and the operating procedure was carried out. After the operation was completed, a blank blood sample was then injected to observe whether there was any carryover of the analytes.

#### 2.6.3 Accuracy and precision

For precision and accuracy experiments, selected blank blood samples spiked with analytes at four levels (LLOQ, low, medium and high) and a certain amount of internal standard substances were subjected to analysis. The accuracy was determined by comparing the analyte concentrations at four levels with the theoretical spiked concentrations. Precision was represented by intra - and inter - day CVs. The intra - day CV was determined by conducting six analyses on each level within 1 day, and the inter - day CV was determined by performing 18 analyses on each level over three consecutive days. The acceptable range of accuracy is within ±15% of the theoretical spiked concentration, except for the LLOQ level, where the acceptable range is within ±20% of the theoretical spiked concentration. The acceptable precision requires the CV to be within ±15%, and for the LLOQ, the CV should be within ±20%.

#### 2.6.4 Matrix effect and extraction recovery

Target analytes at different levels were spiked into the solvent and the extracted blank blood samples (from six different sources) respectively. The matrix effect was evaluated according to the ratio of the peak areas of the components spiked into the blank blood and the solvent. Before and after extraction, target analytes at different levels were spiked into blank blood samples. The extraction recovery was evaluated based on the ratio of the peak areas of the target analytes after extraction to those before extraction.

### 2.7 Stability

#### 2.7.1 Long-term stability

Blood samples obtained from workers exposed to p-NA were stored at a temperature of −80 °C. These samples underwent analysis on the 1st, 5th, 10th, 15th, and 20th days, respectively. The stability of various target analytes within the blood was assessed by examining the extent of their degradation. When the decline rate goes beyond 15%, it is seen as evidence that the sample does not comply with the set criteria.

#### 2.7.2 Short-term stability

Blood samples obtained from workers exposed to p-NA were divided into three portions. Two samples were kept at room temperature, while the other one was stored at 4 °C. The two samples kept at room temperature were analyzed at 0 h and 2 h, respectively, and the sample stored at 4 °C was analyzed at 24 h. The stability of various target analytes within the blood was assessed by examining the extent of their degradation. When the decline rate goes beyond 15%, it is seen as evidence that the sample does not comply with the set criteria.

## 3 Results and discussion

### 3.1 Method development

In the detection of p-NA and its metabolites in blood, the separation and purification of samples plays a crucial role. To explore more effective methods for separation and purification, we conducted research on solid-phase extraction (SPE) and liquid-liquid extraction (LLE) techniques. For the SPE, we selected SPE columns such as C18, HLB, WAX, and WCX to separate and purify the samples. The experimental results showed that in the aqueous solution system, SPE columns like HLB, WAX, and WCX had low extraction recoveries for p-PD and p-AAA (<40%), while the C18 column achieved extraction recoveries of over 90% for all components. However, when the C18 column was used for extracting the target analytes in blood, the matrix in the blood exerted a strong matrix effect on p-PD and p-AAA, as the signals of these two substances were much weaker than those in the aqueous solution system. In particular, p-PD was almost undetectable. This might be attributed to the ion suppression of p-PD and p-AAA caused by co-eluting matrix components (such as proteins or phospholipids). In stark contrast, LLE showed promising potential in preliminary studies. This indicated that LLE may be able to overcome the challenges faced by SPE and provide a more reliable approach for the accurate detection of p-NA and its metabolites in blood.

In order to improve the extraction recovery of the target analytes, we conducted a systematic and in-depth optimization study on the types and volumes of organic solvents used in the extraction process. Given that the target analytes all exhibit strong polar characteristics, we focused the research scope on organic solvents with high polarity and immiscibility with water. Ethyl acetate, dichloromethane, and chloroform were chosen as potential extraction solvents for investigation. The experimental results showed significant differences in extraction recoveries among different solvents. The extraction performance of dichloromethane and chloroform was rather disappointing, with extremely low extraction recoveries. Specifically, for the two target analytes, p-PD and p-AAA, the extraction recoveries were even lower than 10%. In sharp contrast to dichloromethane and chloroform, ethyl acetate demonstrated excellent extraction performance with a high extraction recovery. The extraction recoveries of p-PD and p-AAA exceeded 77%, while the extraction recoveries of p-NA, p-NAA, and 2A5NP were even higher than 90%. Based on these results, we selected ethyl acetate as the extraction solvent.

**FIGURE 2:**
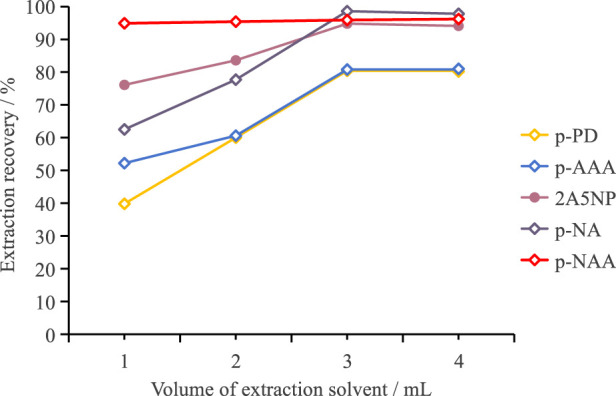
The extraction efficiency of each target analyte at different volumes of the extraction solvent.

After selecting the extraction solvent, we further investigated how the volume of the extraction solvent affected the extraction recovery. We established four distinct volume gradients of the extraction solvent, specifically 1.0 mL, 2.0 mL, 3.0 mL, and 4.0 mL, and then compared the extraction recoveries under each volume condition. Figure 2 presents the variations in the extraction recoveries of each target analyte at different volumes of the extraction solvent. The figure clearly indicates a positive correlation between the extraction recovery of the target analyte and the volume of the extraction solvent. In other words, as the volume of the extraction solvent steadily increased, the extraction efficiency gradually improved. When the volume of the extraction solvent reached 3.0 mL, the extraction efficiency leveled off and no longer showed a significant increase. Therefore, we concluded that the optimal volume of the extraction solvent was 3.0 mL. In addition, increasing the number of extractions may be more effective in improving extraction recovery than simply increasing the volume of the extraction solvent. However, this approach significantly increases the time required for sample preparation, which may be a limiting factor in high-throughput analyses. Moreover, since satisfactory extraction recovery was achieved by increasing the volume of the extraction solvent, we did not evaluate the effect of multiple extractions.

**FIGURE 3:**
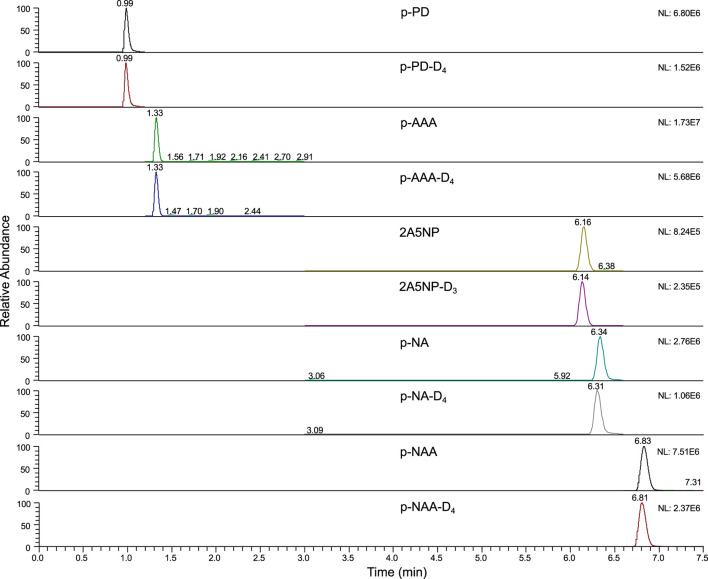
Typical extracted ion chromatographs of standards (50 μg/L).

Among the main determinants for the separation and retention of analytes, the chromatographic column holds a significant place. The ACQUITY UPLC® BEH shield RP18 chromatographic column employs Shield bonded-phase and bridged ethyl hybrid technology. It embeds the carbamate functional group into the alkyl chain of the bonded phase, thereby providing excellent peak profiles for basic analytes. With the UPLC conditions optimized, this column displays high-level column efficiency and peak symmetry. All analytes achieve satisfactory peak profiles and signal intensities, so it was selected as the analytical column (Figure 3). In addition, according to the signal intensity and peak profile, a mobile phase composed of a 0.1% formic acid solution and methanol was selected, and the elution gradient was optimized simultaneously.

### 3.2 Method validation

#### 3.2.1 Linearity and sensitivity

In the concentration range of 1 μg/L - 100 μg/L, these five target analytes showed a good linear relationship. The calibration curves of all analytes had correlation coefficient(*r*) values surpassing 0.999. The difference between the calibrated values and the nominal values of all analytes was within the error range of ±10%. In addition, the LLODs and LLOQs of the target analytes in the blood sample were observed to lie within the intervals of 0.6–2.2 μg/L and 2.0–7.4 μg/L, respectively (Table 2). Amongst these analytes, p - NA had the uppermost LLOD and LLOQ values, while p - PD had the least values for these two key parameters in the analysis.

**TABLE 2:** Correlation coefficient, precision, accuracy, LLOD, and LLOQ.

Compound	Correlation coefficient (*r*)	LLOD (μg/L)	LLOQ (μg/L)	Spiked concentration (μg/L)	Intra-day		Inter-day	
					Accuracy (%, n = 6)	CV (%, n = 6)	Accuracy(%, n = 18)	CV (%, n = 18)
p-PD	0.9998	0.6	2.0	2	89.0	3.2	87.5	3.6
				20	92.5	3.9	94.2	3.9
				225	90.3	4.2	92.3	4.7
				500	92.4	4.6	93.5	5.2
p-AAA	0.9998	0.9	3.1	3	95.6	4.5	94.6	5.4
				20	95.3	3.1	96.7	4.2
				225	90.6	4.3	93.5	5.6
				500	91.1	4.4	94.5	6.9
2A5NP	0.9998	1.4	4.8	5	83.1	9.3	84.2	8.7
				20	91.9	5.6	92.5	5.1
				225	91.8	4.4	92.9	4.0
				500	91.6	3.8	93.1	4.6
p-NA	0.9997	2.2	7.4	7.5	99.9	9.9	95.8	7.8
				20	100.4	7.8	96.8	7.0
				225	93.2	4.3	94.5	5.2
				500	91.6	5.2	92.0	5.4
p-NAA	0.9998	0.9	3.0	3	101.3	3.9	98.5	4.5
				20	99.0	2.2	95.4	3.2
				225	92.7	4.4	93.2	3.9
				500	91.0	3.8	92.1	4.5

#### 3.2.2 Selectivity and carryover

**FIGURE 4:**
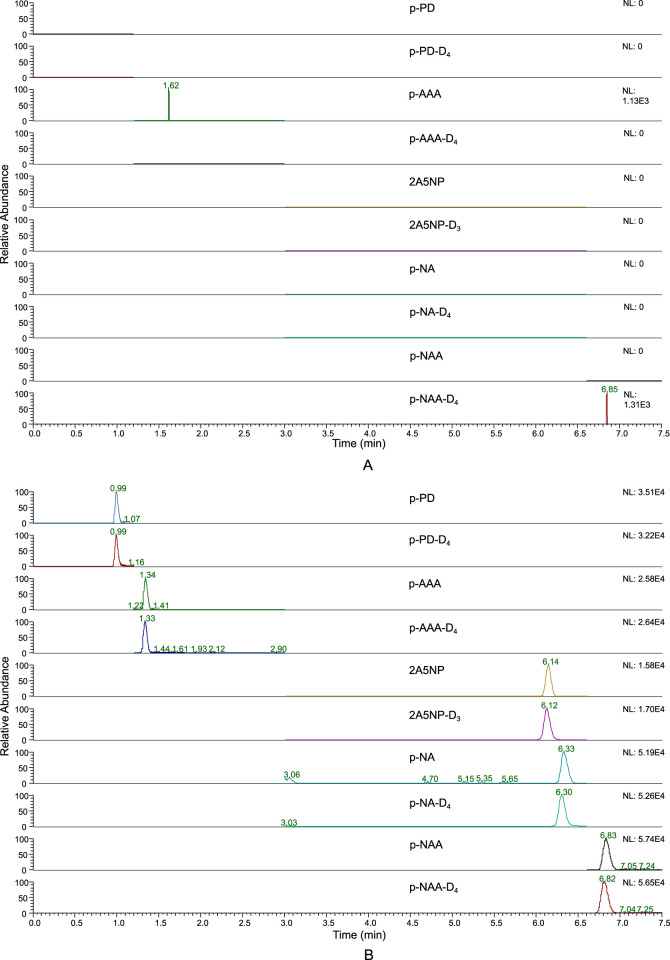
Extracted ion chromatographs of the mixed blank blood sample **(A)** and the LLOQ level matrix - spiked sample **(B)**.

After completing the analysis of the blank blood samples, we observed that there were no interfering peaks within ±5% of the retention time of each target analyte, indicating that the method adopted had good selectivity (Figure 4). After injecting the high-concentration calibration standard and running the program, no target peaks appeared when the blank blood sample was injected again, indicating that the carryover effect was negligible.

#### 3.2.3 Accuracy and precision

Table 2 presents the results of the accuracy and precision of the analytical method. The analytical accuracy of all target analytes in the spiked blood samples at low, medium, and high levels ranged from 90.3% to 100.4% (the accuracy at the LLOQ level ranged from 83.1% to 101.3%). The intra - and inter - day CVs of all target analytes were less than 9.9% and 8.7% respectively. Both the accuracy and precision successfully met the requirements of “Bioanalytical Method Validation-Guidance for Industry” issued by U.S. Department of Health and Human Services Food and Drug Administration (United States Food and Drug Administration, 2018).

#### 3.2.4 Matrix effect and extraction recovery

Tables 3, 4 present the results of matrix effect and extraction recovery respectively. The matrix effects of the five target analytes were between 78.2% and 107.5%, indicating that the matrix effects were weak. In addition, the extraction recoveries of these five target analytes ranged from 77.3% to 99.3%.

**TABLE 3:** Matrix effect for the analytes.

Compound	Spiked concentration (μg/L)	Matrix effect (%)						
		Source						Mean
		1 (n = 5)	2 (n = 5)	3 (n = 5)	4 (n = 5)	5 (n = 5)	6 (n = 5)	
p-PD	10	78.6	78.2	80.0	78.8	75.9	77.5	78.2
	100	78.4	75.6	80.0	81.2	79.2	76.9	78.6
	200	77.7	74.3	79.7	78.5	79.6	81.2	78.5
p-AAA	10	91.8	101.2	103.4	105.2	102.3	101.5	100.9
	100	100.7	106.4	112.8	105.5	107.2	103.9	104.9
	200	103.1	106.9	112.4	110.2	104.5	106.2	107.2
2A5NP	10	94.5	101.8	104.3	98.9	97.8	102.2	99.9
	100	100.4	101.3	104.7	104.5	103.9	102.5	102.3
	200	99.6	99.4	102.6	105.2	98.5	101.2	101.1
p-NA	10	91.7	79.8	79.9	89.5	79.8	83.5	84.0
	100	90.9	80.8	82.7	85.5	83.9	79.8	83.9
	200	89.0	80.2	81.1	82.5	86.5	85.4	84.1
p-NAA	10	100.9	111.8	113.5	102.3	105.6	108.5	107.1
	100	100.9	109.6	112.6	105.6	110.2	105.9	107.5
	200	102.3	104.4	107.4	105.6	102.2	105.4	104.6

**TABLE 4:** Extraction recovery for the analytes.

Compound	Spiked concentration (μg/L)	Extraction recovery (%, n = 6)
p-PD	20	77.5
	225	80.1
	500	83.7
p-AAA	20	77.3
	225	82.9
	500	82.9
2A5NP	20	88.8
	225	97.1
	500	98.5
p-NA	20	99.3
	225	98.7
	500	97.9
p-NAA	20	92.8
	225	97.8
	500	97.2

### 3.3 Stability

#### 3.3.1 Long-term stability

A decline rate exceeding 15% is regarded as a sign that the sample does not meet the stability requirements. The decline rates of all target analytes in the samples on the 20th day were below 15%. This result clearly indicates that blood samples are capable of remaining stable for no less than 20 days when stored at - 80 °C.

#### 3.3.2 Short-term stability

A decline rate exceeding 15% is considered as a sign that the sample does not meet the stability requirements. After being kept at room temperature for 2 h and at 4 °C for 24 h, the decline rate of all target analytes was less than 15%. This result clearly indicates that the target analytes in the blood samples have good short - term stability.

### 3.4 Method application

To verify the applicability of the established method, 15 blood samples were selected for quantitative analysis of the concentrations of p-NA and its metabolites. These samples cover research subjects with different exposure backgrounds. Among them, 10 samples were from workers (aged between 37 and 56, with a male-to-female ratio of 4:1) who had been exposed to p-NA in their occupational environment for a long time, and 5 samples were collected from administrators (aged between 35 and 52, with a male-to-female ratio of 3:2) who had never been exposed to p - NA.

In the detection of blood samples from workers exposed to p-NA, significant differences in the concentrations of substances were observed (Table 5). Specifically, the concentration range of p-NA spanned widely, from <LLOQ to 86.4 μg/L, indicating that there were obvious differences in the doses of p-NA absorbed by different individuals during actual exposure. As a product of the nitro reduction of p-NA, the concentration range of p-PD was from <LLOQ to 7.2 μg/L, reflecting the transformation of p-NA in the *in vivo* metabolic process. As one of the products of amino acetylation, the concentration of p-AAA fluctuated between < LLOQ and 6.8 μg/L; the concentration range of 2A5NP was from <LLOQ to 26.4 μg/L; and the concentration of p-NAA was from <LLOQ to 7.9 μg/L. The detection of the concentrations of these substances demonstrated the effectiveness of this detection method in capturing trace substances. In sharp contrast, p-NA and its metabolites were not detected in the blood samples of the 5 administrators who had no exposure to p-NA. This result further highlights the specificity and accuracy of the detection method, which can effectively distinguish the characteristics of blood samples from exposed and unexposed populations.

**TABLE 5:** Concentrations of target analytes in the blood samples.

Sample ID		Concentration (μg/L)				
		p-NA	p-PD	p-AAA	2A5NP	p-NAA
Exposure to p-NA	1	25.3	2.0	<LLOQ	10.3	<LLOQ
	2	35.6	2.8	<LLOQ	11.8	3.2
	3	38.9	3.1	<LLOQ	14.5	3.5
	4	86.4	7.2	6.8	26.4	7.9
	5	<LLOQ	<LLOQ	<LLOQ	<LLOQ	<LLOQ
	6	12.5	<LLOQ	<LLOQ	5.9	<LLOQ
	7	9.9	<LLOQ	<LLOQ	2.8	<LLOQ
	8	75.6	6.0	4.7	2.9	6.9
	9	69.8	5.5	4.4	4.9	6.3
	10	22.3	<LLOQ	<LLOQ	9.2	<LLOQ
Non-exposure to p-NA	11	<LLOQ	<LLOQ	<LLOQ	<LLOQ	<LLOQ
	12	<LLOQ	<LLOQ	<LLOQ	<LLOQ	<LLOQ
	13	<LLOQ	<LLOQ	<LLOQ	<LLOQ	<LLOQ
	14	<LLOQ	<LLOQ	<LLOQ	<LLOQ	<LLOQ
	15	<LLOQ	<LLOQ	<LLOQ	<LLOQ	<LLOQ

The results show that whether it is the accurate determination of trace target substances in the blood of p-NA exposed populations or the effective exclusion of interfering factors in the blood samples of unexposed populations, the established detection method exhibits good analytical performance. Therefore, we believe that this method can be applied to the quantitative detection of p-NA and its metabolites in actual blood samples.

## 4 Conclusion

In this study, a novel analytical method was successfully developed, which was based on the UPLC-Q-Orbitrap HRMS technology and incorporated a simple LLE procedure. This method can simultaneously quantify the contents of p-NA and its metabolites (p-PD, p-AAA, 2A5NP, p-NAA) in blood. In this method, HRMS plays a crucial role. It has excellent resolution, a characteristic that enables a more precise qualitative determination of target analytes, greatly improving the accuracy of qualitative results. In addition, the electrostatic field orbitrap mass analyzer used ensures the performance of the method in quantitative analysis. Its quantitative ability is on par with that of triple-quadrupole mass spectrometry. After verification, the newly developed method demonstrated satisfactory precision, accuracy, and sensitivity in the detection of p-NA and its metabolites in blood. This method provides solid methodological support for the internal exposure assessment of p-NA, contributing to the accurate evaluation of the actual human exposure to p-NA. Meanwhile, it also offers methodological support for subsequent metabolic studies of p - NA in the body.

## Data Availability

The original contributions presented in the study are included in the article; further inquiries can be directed to the corresponding author.

## References

[B1] Abou-Elwafa Abdallah M. Nguyen K. Ebele A. J. Atia N. N. Ali H. R. H. Harrad S. (2019). A single run, rapid polarity switching method for determination of 30 pharmaceuticals and personal care products in waste water using Q-Exactive Orbitrap high resolution accurate mass spectrometry. J. Chromatogr. A 1588, 68–76. 10.1016/j.chroma.2018.12.033 30587347

[B2] American Conference of Governmental Industrial Hygienists (2021). TLVs® and BEIs®: threshold limit values for chemical substances and physical agents & biological exposure indices. Cincinnati: American Conference of Governmental Industrial Hygienists.

[B3] Bakdash A. A. Ganswindt M. Herre S. Nadulski T. Pragst F. (2006). Lethal poisoning with p-nitroaniline. Toxichem Krimtech 73, 61–65.

[B4] Bronaugh R. L. Maibach H. I. (1985). Percutaneous absorption of nitroaromatic compounds: *in vivo* and *in vitro* studies in the human and monkey. J. Invest. Dermatol. 84 (3), 180–183. 10.1111/1523-1747.ep12264716 3919108

[B5] Chai Y. Qu D. Ma D. Chen W. Huang S. (2018). Carbon quantum dots/Zn^2+^ ions doped-CdS nanowires with enhanced photocatalytic activity for reduction of 4-nitroaniline to p-phenylenediamin. Appl. Surf. Sci. 450, 1–8. 10.1016/j.apsusc.2018.04.121

[B6] Enders J. R. Weed R. A. Griffith E. H. Muddiman D. C. (2022). Development and validation of a high resolving power absolute quantitative per- and polyfluoroalkyl substances method incorporating Skyline data processing. Rapid Commun. Mass Sp. 36 (11), e9295. 10.1002/rcm.9295 35275435 PMC9287086

[B7] French C. L. Yaun S. S. Baldwin L. A. Leonard D. A. Zhao X. Q. Calabrese E. J. (1995). Potency ranking of methemoglobin-forming agents. J. Appl. Toxicol. 15 (3), 167–174. 10.1002/jat.2550150306 7560736

[B8] Gu C. Bao Y. (2011). Investigation and disposal of an acute p - nitroaniline poisoning incident. Chin. For. Med. Res. 9 (35), 80. 10.3969/j.issn.1674-6805.2011.35.060

[B9] Huajing Industrial Research Institute (2021). Investment analysis and development strategy consultation report on China's p-nitroaniline industry from 2021 to 2026.

[B10] Li Z. Li Y. Chen W. Cao Q. Guo Y. Wan N. (2017). Integrating MS1 and MS2 scans in high-resolution parallel reaction monitoring assays for targeted metabolite quantification and dynamic ^13^C-labeling metabolism analysis. Anal. Chem. 89 (1), 877–885. 10.1021/acs.analchem.6b03947 27966897

[B11] Ma H. Wang M. Pu C. Zhang J. Zhao S. Yao S. (2009). Transient and steady-state photolysis of p-nitroaniline in aqueous solution. J. Hazard. Mater. 165, 867–873. 10.1016/j.jhazmat.2008.10.077 19062165

[B12] Ministry of Environmental Protection of the People's Republic of China (2009). National list of environmental health risks of pollutants—Part 1 chemicals. Beijing: China Environmental Science Press.

[B13] National Health and Family Planning Commission of the People's Republic of China (2017). General principles of biological monitoring method in occupational population, GBZ/T 295-2017. Beijing: Standards Press of China.

[B14] National Health Commission of the People’s Republic of China (2019). Occupational exposure limits for hazardous agents in the workplace - Part 1: chemical hazardous agents, GBZ 2.1-2019. Beijing: Standards Press of China.

[B15] Qi H. Feng F. Zhai J. Chen F. Liu T. Zhang F. (2019). Development of an analytical method for twelve dioscorea saponins using liquid chromatography coupled to Q-Exactive high resolution mass spectrometry. Talanta 191, 11–20. 10.1016/j.talanta.2018.08.040 30262039

[B16] Sun J. Sun S. Fan M. Guo H. Qiao L. Sun R. (2007). A kinetic study on the degradation of p-nitroaniline by Fenton oxidation process. J. Hazard. Mater. 148, 172–177. 10.1016/j.jhazmat.2007.02.022 17379403

[B17] United States Food and Drug Administration (2018). Bioanalytical method validation guidance for industry. U. S. Food Drug Adm. Available online at: http://www.fda.gov/AnimalVeterinary/GuidanceComplianceEnforcement/GuidanceforIndustry/default.htm (Accessed May 18, 2025).

[B18] Xia Y. (1991). Encyclopedia of chemical substances toxicity. Shanghai: Shanghai Scientific and Technological Literature Press.

[B19] Zhang G. Xie X. Wang L. (2006). Investigation into an accident of p - nitroaniline poisoning. Zhejiang Prev. Med. 18 (4), 41–42. 10.3969/j.issn.1007-0931.2006.04.028

[B20] Zhao Y. Bai X. Song T. Zhang G. Yuan Y. Liu Y. (2019). Determination of environmental estrogens and bisphenol A in water samples by ultra-high performance liquid chromatography coupled to Q-Exactive high resolution mass spectrometry after magnetic solid-phase extraction. Microchem. J. 151, 104212. 10.1016/j.microc.2019.104212

